# Risk Factors for Incidence of Cardiovascular Diseases and All-Cause Mortality in a Middle Eastern Population over a Decade Follow-up: Tehran Lipid and Glucose Study

**DOI:** 10.1371/journal.pone.0167623

**Published:** 2016-12-08

**Authors:** Mahsa Sardarinia, Samaneh Akbarpour, Mojtaba Lotfaliany, Farideh Bagherzadeh-Khiabani, Mohammadreza Bozorgmanesh, Farhad Sheikholeslami, Fereidoun Azizi, Farzad Hadaegh

**Affiliations:** 1 Prevention of Metabolic Disorders Research Center, Research Institute for Endocrine Sciences, Shahid Beheshti University of Medical Sciences, Tehran, Iran; 2 Department of Epidemiology & Biostatistics, School of Public Health, Tehran University of Medical Sciences, Tehran, Iran; 3 Non-Communicable Disease Control, School of Population and Global Health, University of Melbourne, Victoria, Australia; 4 Endocrine Research Center, Research Institute for Endocrine Sciences, Shahid Beheshti University of Medical Sciences, Tehran, Iran; Children's National Health System, UNITED STATES

## Abstract

**Background:**

To examine the association between potentially modifiable risk factors with cardiovascular disease (CVD) and all-cause mortality and to quantify their population attributable fractions (PAFs) among a sample of Tehran residents.

**Methods:**

Overall, 8108 participants (3686 men) aged≥30 years, were investigated. To examine the association between risk factors and outcomes, multivariate sex-adjusted Cox proportional hazard regression analysis were conducted, using age as time-scale in two models including general/central adiposity: 1)adjusted for different independent variables including smoking, education, family history of CVD and sex for both outcomes and additionally adjusted for prevalent CVD for all-cause mortality 2)further adjusted for obesity mediators (hypertension, diabetes, lipid profile and chronic kidney disease). Separate models were used including either general or central adiposity.

**Results:**

During median follow-up of >10 years, 827 first CVD events and 551 deaths occurred. Both being overweight (hazard ratio (HR), 95%CI: 1.41, 1.18–1.66, PAF 13.66) and obese (1.51, 1.24–1.84, PAF 9.79) played significant roles for incident CVD in the absence of obesity mediators. Predicting CVD, in the presence of general adiposity and its mediators, significant positive associations were found for hypercholesterolemia (1.59, 1.36–1.85, PAF 16.69), low HDL-C (1.21, 1.03–1.41, PAF 12.32), diabetes (1.86, 1.57–2.27, PAF 13.87), hypertension (1.79, 1.46–2.19, PAF 21.62) and current smoking (1.61, 1.34–1.94, PAF 7.57). Central adiposity remained a significant positive predictor, even after controlling for mediators (1.17, 1.01–1.35, PAF 7.55). For all-cause mortality, general/central obesity did not have any risk even in the absence of obesity mediators. Predictors including diabetes (2.56, 2.08–3.16, PAF 24.37), hypertension (1.43, 1.11–1.84, PAF 17.13), current smoking (1.75, 1.38–2.22, PAF 7.71), and low education level (1.59, 1.01–2.51, PAF 27.08) were associated with higher risk, however, hypertriglyceridemia (0.83, 0.68–1.01) and being overweight (0.71, 0.58–0.87) were associated with lower risk.

**Conclusions:**

Modifiable risk factors account for more than 70% risk for both CVD and mortality events.

## Introduction

Cardiovascular diseases (CVD) are the leading cause of both mortality and disability worldwide [1]. Approximately, two-thirds (63%) of premature deaths in adults (aged 15–69 years), and three-out-of-four of all adult deaths are attributable to non-communicable diseases, which are mainly due to CVD [2]. It has been predicted that by 2030, over 23.3 million people will die annually from CVD [3], most of which will occur in low-and-middle income countries, such as those in Middle East, where rapid changes in lifestyle, ageing populations and transforming environments all contribute to the dramatic pace of the epidemic [4]. It has been shown that CVDs are becoming a major health problem in this region mostly due to the already high and fast increasing prevalence of cardiovascular risk factors [5–11]. In spite of having higher CVD morbidity and mortality; compared to Western countries, data in regards to the dynamics of CVD from Eastern population is limited [12].

Management of the multiple modifiable risk factors associated with incident CVD and all-cause mortality in the general populations is an ongoing challenge for primary health care decision makers. Population attributable fraction (PAF), a measure of potential global impact, is one of the most applicable indices in public health which can assist policymakers in prioritizing health strategies among the general population [13]. It is the hypothetical reduction in incidence that would be observed if the population were entirely unexposed, compared with its current (actual) exposure pattern [13].

The aim of the current study is to determine the associations of potentially modifiable cardiovascular risk factors including general or central obesity, smoking, educational level, blood pressure categories, glucose intolerance status, lipid profile and chronic kidney disease (CKD) with CVD and all-cause mortality and their PAF, using data from a long-term population–based prospective study, the Tehran Lipid-Glucose Study (TLGS), with over a decade long follow-up.

## Methods and Materials

### Study design and sample

The Tehran Lipid and Glucose Study (TLGS) is an ongoing prospective population-based study performed on a representative sample of the Tehran population, aimed at determining the prevalence and incidence of non-communicable diseases and their risk factors. Details of the TLGS have been reported elsewhere [14]. In brief, one baseline examination (1999–2001) and 3 follow-up phases at about 3-year intervals had been carried out until 2012.

### Study population

**Fig 1:**
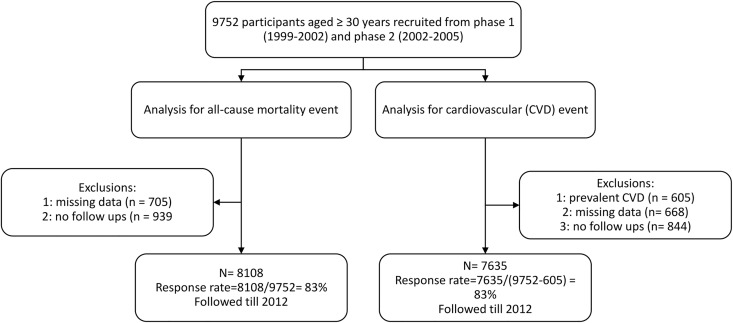
Study population.

Exclusions were carried out at two separate lines for all-cause mortality and CVD event analysis. For all-cause mortality analysis, from among 9752 participants (5331 women and 4421 men) ≥30 years [7550 people from the baseline examination (1999–2001) and 1597 new participants were recruited from the second phase (2001–2005)], after exclusions of 705 cases with missing data on any examined baseline variables and 939 participants with no follow-up from baseline examination; finally 8108 participants (4422 women and 3686 men) remained to be followed till 2012, (mean follow-up of 10.67 years and 3.88 interquartile range, response rate 83%) (Fig 1).

For CVD event analysis, among 9752 participants aged 30 years or more were selected. Subjects with prevalent CVD on baseline examination (N = 605), missing data (N = 668) or without any follow-up (N = 844) were excluded, leaving 7635 participants (4205 women and 3430 men) for the current study, who were followed up till 2012 (mean follow-up of 10.73 years and 3.65 interquartile range, response rate 83%) (Fig 1).

A written consent was obtained from all participants after being informed about the general aspects of the investigation and the study protocol conforms to the ethical guidelines of the 1975 Declaration of Helsinki as reflected in prior approval by the ethical committee of the Research Institute for Endocrine Sciences (RIES).

### Clinical and laboratory measurements

Using a standard questionnaire, a trained interviewer collected information, which included demographic data, drug history and family history of type 2 diabetes (T2D) and CVD. Details of anthropometric measurements including weight, height and waist circumference (WC), systolic blood pressure (SBP) and diastolic blood pressure (DBP) measurements have been reported elsewhere (14).

A blood sample was taken between 7:00 and 9:00 AM from all study participants, after 12 to 14 hours overnight fasting. Details of laboratory measurement including fasting plasma glucose (FPG), total cholesterol (TC), triglyceride (TG) and high-density lipoprotein (HDL-C) were reported elsewhere (14).

Glomerular filtration rate (GFR) was estimated using the abbreviated prediction equation, provided by the Modification of Diet in Renal Disease (MDRD) study as follows:

$$GFR=186\times {\left(SCr\right)}^{\text{-}1.145}\times {\left(Age\right)}^{\text{-}0.203}\times \left(0.742\;if\;female\right)\times \left(1.210\;if\;African\;\text{-}\;American\right)$$

In this equation, estimated GFR (eGFR) is expressed as mL/min per 1.73 m2 and serum creatinine (Scr) as mg/dL [15].

### Definition of terms and outcomes measurement

Cardiovascular risk factors were categorized and defined as illustrated in Table 1. For a collection of cardiovascular outcomes, each participant has followed up annually for any medical event; a trained nurse asked them regarding any related medical condition, and a trained physician collected complementary data for that event, during a home visit, and also by the acquisition of data from medical files from hospitals and any medical encounters. In the current study, the events targeted were the first CVD event, including definite myocardial infarction (MI), probable MI, unstable angina, angiographic-proven CHD, and stroke (defined by a new neurological deficit that lasted more than 24 h). In case of mortality, data were collected by an authorized local physician from the hospital or the death certificate. Data collected were evaluated by an outcome committee (Cohort Outcome Panel) consisting of a principal investigator, an internist, an endocrinologist, a cardiologist, an epidemiologist, and the physician who collected the outcome data; other experts were invited as required for evaluation of non-communicable disorders. The final diagnosis was by consensus of the majority of committee members (i.e. by ≥3 members of the committee).

**Table 1:** Definition of different cardiovascular risk factors categories; Tehran Lipid and Glucose Study 1999–2012

CVD risk factor	Definition
**Glucose intolerance**	
Non-diabetic	FPG< 100mg/dl (5.55 mmol/l) and 2h-PCPG<140 mg/dl (7.77 mmol/l) and taking no anti-diabetic medications
Diabetic[16]	FPG ≥126 mg/dl (7 mmol/l) or 2 h-PCPG ≥200 mg/dl (11.1 mmol/l) or taking any medication for T2D
Pre-diabetic[17]	Individuals who were not characterized as non-diabetic or diabetic
**General obesity**	
Normal BMI	BMI<25 kg/m^2^
Overweight	25≤ BMI<30 kg/m^2^
Obese	BMI ≥ 30 kg/m^2^
**Central obesity**	Waist circumference ≥95 cm [18]
**Blood pressure [19]**	
Normotensive	SBP <120 mmHg and DBP <80 mmHg
Hypertension	SBP ≥140 mmHg or DBP ≥90 mmHg or taking any medication for hypertension
Pre-hypertensive	Individuals who were not characterized as normotensive or hypertensive
**Dyslipidemia [20]**	
Hypercholesterolemia	TC ≥ 6.21 mmol/L or using lipid lowering medications
Hypertriglyceridemia	TG ≥ 1.69 mmol/L
Low HDL-C	HDL-C< 1.06 mmol/L (men) or HDL-C<1.29 mmol/L (women)
**Family history of CVD**	History of myocardial infarction, stroke or sudden cardiac death in a male first degree relative <55 years or in a female first degree relative <65
**Current smoking**	Participants who used any tobacco product (cigarette, pipe, and water pipe) at time of examination
**Education**	
<6	Illiterate participant and those with primary school education or less (less than 6 years of education)
6–12	Those who had a diploma or did not complete the diploma but finished the primary school (6 years)
>12	Those with higher than diploma education(more than 12 years of education)
**CKD**	Those with eGFR<60 mL/min/1.73 m^2^[21].

FPG, Fasting plasma glucose; 2h-PCPG, 2 hour-post-challenge plasma glucose; BMI, body mass index; T2D, type 2 diabetes; SBP, systolic blood pressure; DBP, diastolic blood pressure; TC, total cholesterol; TG, triglycerides; HDL-C, high-density lipoprotein cholesterol; CKD, chronic kidney disease; eGFR, estimated glomerular filtration rate.

### Statistical analysis

Baseline characteristics of the study population are presented as mean or frequency (percentage) for continuous and categorical variables, respectively.

All possible first-order interactions between sex and independent variables were checked in multivariable analysis (i.e. sex × BMI, sex × glucose intolerance status, sex × WC, sex × blood pressure categories, sex × TC, sex × HDL-C, sex× TG, sex × family history of CVD, sex × smoking, sex × education levels, sex × CKD, sex × prevalent CVD). Considering the Bonferroni correction as a multiple comparison correction, we found no P value ≤ 0.01 for interaction between different risk factors with sex for either CVD or mortality events [22]; hence, we adjusted for sex, to reach full statistical power, in the different models. Similarly, we also found no interaction between prevalent CVD and different risk factors for mortality events (all P-values for interactions >0.01). All of the interactions were tested by log-likelihood ratio test.

Cox proportional hazard models with age as the time scale [23], were used to estimate the hazard ratios (HRs) with 95% confidence intervals (95% CIs) of different risk factors for development of each outcome of interest in two models including general/central obesity adjusted for 1) different independent variables including smoking, education, family history of CVD and sex for both outcomes and additionally adjusted for prevalent CVD for all-cause mortality, 2) the above risk factors plus obesity mediators including blood pressure categories, glucose intolerance status, abnormal lipid profile (hypercholesterolemia, low HDL-C, and hypertriglyceridemia) [24] and chronic kidney disease (CKD) [25–28], applying the enter method. Non-modifiable risk factors including sex, family history of CVD and prevalent CVD were considered as confounder variables in our data analysis. To overcome the issue of collinearity, we conducted separate models for general and central adiposity.

The proportional hazard assumption was examined using Schoenfeld residuals test and plotting log [–log (survival)] versus log (time) to assess parallelism. Follow-up duration was defined as the period between entrance to study and the end point; end points were considered as the first CVD event or mortality, and censoring was defined as leaving the residence area, lost to follow-up or until the end of the study.

PAFs were calculated using the following formula [29]:

$$PAF=P_{C}\left(\frac{HR_{adj}-1}{HR_{adj}}\right)$$

HR_adj_ indicates multivariate-adjusted hazard ratio for a particular exposure factor and P_c_ represents the prevalence of that exposure among individuals with the outcome of interest. Stata (Stata Corp 12 SE) was used for data analysis and p values<0.05 were considered statistically significant. Additionally, to recover our missing data, we used *multivariate imputation by chained equation*, using a regression including all of the variables in the main models [30, 31].

## Results

The study sample for analysis of all-cause mortality consisted of 8108 participants, aged ≥30 years, mean age 47.52 (range 30–88 years). Baseline characteristics of respondent and non-respondent participants (those with missing data at the baseline or with no follow-up data) in our sample are shown in Table 2. Compared to non-respondents, respondents were older, more educated and hypercholesterolemic and had higher WC; however, they had lower BMI, the rates of current smoking as well as prevalent CVD. Aforementioned results were similar in study sample for CVD events (n = 7635).

**Table 2:** Baseline characteristics* of respondent and non-respondent participants in both genders; Tehran Lipid and Glucose Study 1999–2005.

		Respondent (n = 8108)	Non-respondent (n = 1644)	P-value†
**Continuous variables:**		Mean (SD)	Mean (SD)	
	Age (years) **	47.52(12.44)	48.30(13.96)	0.025
	BMI (kg/m^2^)	27.52(4.56)	27.18(4.74)	0.012
	Waist circumference (cm)	90.87(11.53)	89.87(11.52)	0.004
	Triglyceride levels (mmol/L)	2.06(1.34)	2.12(1.68)	0.170
	Total cholesterol (mmol/L)	5.53(1.18)	5.57(1.22)	0.226
	HDL-C (mmol/L)	1.07(0.28)	1.08(0.29)	0.066
	Systolic blood pressure (mmHg)	121.69(19.96)	122.37(21.13)	0.239
	Diastolic blood pressure (mmHg)	78.64(11.03)	78.68(11.06)	0.900
	Fasting plasma glucose (mmol/L)	5.62(1.96)	5.67(2.29)	0.331
	eGFR (mL/min/1.73 m2)	68.89(11.54)	68.03(12.55)	0.018
**Categorical variables††:**				
	Sex(men)	3686(45.46)	735(44.71)	0.576
	General obesity			
	Normal BMI	2424(29.90)	437(26.58)	0.076
	Overweight	3524(43.46)	579(35.22)	
	Obese	2160(26.64)	326(19.83)	
	Central obesity	3047(37.58)	466(28.35)	0.057
	Hypertriglyceridemia	4328(53.38)	693(42.15)	0.109
	Hypercholesterolemia	2248(27.73)	423(25.73)	0.015
	Low HDL-C	5701(70.31)	958(58.27)	0.442
	Blood Pressure			
	Normal	3279(40.44)	564(34.31)	0.728
	Pre hypertension	2679(33.04)	481(29.26)	
	Hypertension	2150(26.52)	389(23.66)	
	Blood Sugar Status			
	Non-diabetic	5096(62.85)	680(41.4)	0.003
	Pre-diabetic	1862(22.97)	266(16.2)	
	Diabetic	1150(14.18)	205(12.5)	
	CKD**	1698(20.94)	327(19.89)	0.09
	Current smoking	1356(16.72)	299(18.18)	<0.0001
	Education status			
	>12 years	988(12.19)	186(11.31)	0.022
	6–12 years	3789(46.73)	713(43.37)	
	<6 years	3331(41.08)	729(44.34)	
	Family history of CVD	1358(16.75)	255(15.51)	0.618
	Anti-hypertensive drug	776(9.57)	171(10.40)	0.300
	Anti-diabetic drugs	447(5.51)	90(5.47)	0.950
	Lipid lowering drugs	327(4.03)	82(4.99)	0.078
	Prevalent CVD	472(5.82)	133(8.09)	0.001

* Baseline characteristics were reported by frequency (percent) in each category and compared by Chi-square test otherwise indicated.

** Age was reported by mean (SD) in each group and compared by Student’s independent t-test.

† P-values were derived from comparison between respondent and non-respondent participants

††for categorical variables, numbers (percent), the definition of categorical variables is shown in Table 1.

eGFR, glomerular filtration rate; HDL-C, high-density lipoprotein cholesterol; CKD, chronic kidney disease.

During the follow-up period, 827 first CVD events and 551 deaths occurred. Underlying causes of mortality were CVD (n = 255), cancer (n = 89), sepsis and pneumonia (n = 48), accidents (n = 19), other heart diseases (n = 23), diabetes complications (n = 18), unknown (n = 53) and miscellaneous reasons (n = 46).

Multivariate-adjusted hazard ratios and 95% confidence intervals (CIs) of potential cardiovascular risk factors as well as their PAFs for incident CVD and all-cause mortality, controlled for BMI and in the absence of obesity mediators are shown in Table 3. Accordingly, both being overweight (HR, 95% CI: 1.41, 1.18–1.66) and obese (1.51, 1.24–1.84) played significant roles for in a higher risk of incident CVD. Furthermore, there were significant positive associations between family history of CVD, smoking and male gender with CVD events. Furthermore, smoking, less than 6 years of education, family history of CVD, male gender, and prevalent CVD remained significant predictors for higher risk of mortality. Our results showed that being overweight was associated with lower risk of mortality events (0.8, 0.66–0.97, P = 0.030).

**Table 3:** Multivariate-adjusted hazard ratios and 95% confidence intervals (CIs) of potential cardiovascular risk factors for incident CVD and all-cause mortality, without obesity mediators controlling for general adiposity status, Tehran lipid and Glucose study (1999–2012)

		CVD event							All-cause mortality						
		HR	95% confidence interval	p-value	Prevalence*	95% confidence interval	PAF**	95% confidence interval	HR	95% confidence interval	p-value	Prevalence*	95% confidence interval	PAF**	95% confidence
BMI															
	Normal	1			0.24	0.21–0.27	-		1			0.36	0.31–0.39	-	
	Overweight	1.41	1.18–1.66	<0.0001	0.47	0.43–0.50	13.66	6.55–19.87	0.80	0.66–0.97	0.030	0.39	0.35–0.43	-9.75	(-18.03)-(-1.32)
	Obesity	1.51	1.24–1.84	<0.0001	0.29	0.25–0.32	9.79	4.83–14.60	0.98	0.77–1.23	0.878	0.25	0.21–0.29	-	
Current Smoker		1.53	1.27–1.83	<0.0001	0.20	0.17–0.23	6.92	3.61–10.43	1.63	1.29–2.06	<0.0001	0.18	0.14–0.21	6.95	3.14–10.80
Education															
	>12 years	1							1			0.05	0.02–0.06	-	
	6–12 years	1.22	0.92–1.62	0.159			-		1.49	0.94–2.38	0.088	0.22	0.19–0.26	7.23	(-1.21)-15.07
	<6 years	1.16	0.87–1.55	0.295			-		1.67	1.06–2.63	0.027	0.73	0.69–0.77	29.28	3.90–47.72
Family history of CVD		1.58	1.33–1.87	<0.0001	0.21	0.17–0.23	7.70	4.21–10.70	1.33	1.06–1.66	0.011	0.18	0.14–0.21	4.46	0.79–8.34
Male gender (female as a reference)		1.73	1.47–2.02	<0.0001			-		1.42	1.17–1.73	<0.0001			-	
Prevalent CVD							-		2.01	1.62–2.47	<0.0001	0.21	0.17–0.21	10.55	6.50–12.49

*Prevalence represents the prevalence of the exposure among individuals with outcome of interest

**The population attributed fractions were calculated using the following formula: Prevalence*((Hazard ratio-1)/Hazard ratio)

HR, hazard ratio; HDL-C, high-density lipoprotein cholesterol; CKD, chronic kidney disease

The definition of categorical variables is shown in Table 1.

Multivariate-adjusted hazard ratios and 95% confidence intervals (CIs) of potential cardiovascular risk factors as well as their PAFs for incident CVD and all-cause mortality, controlled for BMI and in the presence of obesity mediators are shown in Table 4. Accordingly, significant positive associations between hypercholesterolemia, low HDL-C level, hypertension, diabetes, current smoking, family history of CVD, male gender, and incident CVD were observed (all p values < 0.05). Predicting all-cause mortality, hypertension (1.43, 1.11–1.84), diabetes (2.56, 2.08–3.16) smoking (1.75, 1.38–2.22), less than 6 years of education (1.59, 1.01–2.51), male gender (1.48, 1.21–1.82), prevalent CVD (1.63, 1.32–2.02), family history of CVD (1.24, 0.99–1.55, P = 0.054) revealed positive associations with higher risk of death. However, general obesity (0.79, 0.62-.1.00, P = 0.058), being overweight (0.71, 0.58-.87, P = 0.001) and hypertriglyceridemia (0.83, 68–1.01; P = 0.066) were associated with lower risks of all-cause mortality.

**Table 4:** Multivariate-adjusted hazard ratios and 95% confidence intervals (CIs) of potential cardiovascular risk factors for incident CVD and all-cause mortality, with obesity mediators (diabetes, hypertension, lipid profile and CKD), controlling for general adiposity status, Tehran lipid and Glucose study (1999–2012)

		CVD event								All-cause mortality							
		N (event)	HR	95% confidence interval	p-value	Prevalence*	95% confidence interval	PAF**	95% confidence interval	N (event)	HR	95% confidence interval	p-value	Prevalence*	95% confidence interval	PAF**	95% confidence interval
BMI																	
	Normal	202	1							196	1			0.36	0.31–0.39		
	Overweight	386	1.15	0.96–1.37	0.122					218	0.71	0.58–0.87	0.001	0.39	0.35–0.43	-15.93	(-25.34)-(-6.42)
	Obesity	239	1.06	0.86–1.31	0.550					137	0.79	0.62–1.01	0.058	0.25	0.21–0.28	-6.64	(-12.87)-(0.27)
High TG		540	1.02	0.86–1.20	0.809					295	0.83	0.68–1.01	0.066	0.53	0.49–0.57	-10.85	(-0.23)-(0.56)
High TC		373	1.59	1.36–1.85	<0.0001	0.45	0.41–0.48	16.69	10.85–22.05	210	1.03	0.85–1.25	0.735				
low HDL-C		584	1.21	1.03–1.41	0.020	0.71	0.67–0.73	12.32	1.95–21.22	336	0.94	0.78–1.13	0.560				
Blood pressure status																	
	Normal	174	1			0.21	0.18–0.23			105	1			0.20	0.15–0.22		
	prehypertension	241	1.12	0.92–1.37	0.251	0.29	0.26–0.32			129	0.91	0.70–1.20	0.536	0.23	0.19–0.26		
	Hypertension	412	1.79	1.46–2.19	<0.0001	0.49	0.46–0.53	21.62	14.49–28.79	317	1.43	1.11–1.84	0.005	0.57	0.53–0.61	17.13	5.25–27.84
Blood sugar status																	
	Normal	353	1			0.43	0.39–0.46			200	1			0.36	0.32–0.40		
	Pre diabetes	223	1.11	0.94–1.32	0.214	0.27	0.23–0.29			130	1.13	0.90–1.42	0.267	0.23	0.20–0.27		
	Diabetes	251	1.86	1.57–2.22	<0.0001	0.30	0.27–0.33	13.87	9.80–18.13	221	2.56	2.08–3.16	<0.0001	0.40	0.36–0.44	24.37	18.69–30.07
CKD		276	1.02	0.86–1.19	0.84					233	1.02	0.84–1.23	0.809				
Current Smoker		168	1.61	1.34–1.94	<0.0001	0.20	0.17–0.23	7.57	4.31–11.14	100	1.75	1.38–2.22	<0.0001	0.18	0.14–0.21	7.71	3.85–11.54
Education																	
	>12 years	59	1							21	1			0.05	0.02–0.06		
	6–12 years	269	1.19	0.90–1.58	0.214					124	1.47	0.92–2.33	0.099	0.22	0.19–0.26	7.03	-1.65–14.84
	<6 years	499	1.13	0.85–1.51	0.390					406	1.59	1.01–2.51	0.044	0.73	0.69–0.77	27.08	0.68–46.32
Family history of CVD		171	1.52	1.28–1.80	<0.0001	0.21	0.18–0.23	7.18	3.93–10.22	99	1.24	0.99–1.55	0.054	0.18	0.14–0.21	3.48	(-0.14)-7.45
Male gender(female as a reference)		490	2.02	1.71–2.39	<0.0001					340	1.48	1.21–1.82	<0.0001				
Prevalent CVD		-								117	1.63	1.32–2.02	<0.0001	0.21	0.17–0.21	8.12	4.12–10.60

*Prevalence represents the prevalence of the exposure among individuals with outcome of interest

**The population attributed fractions were calculated using the following formula: Prevalence*((Hazard ratio-1)/Hazard ratio)

HR, hazard ratio; HDL-C, high-density lipoprotein cholesterol; CKD, chronic kidney disease

The definition of categorical variables is shown in Table 1.

PAFs calculations, in CVD event analysis, showed that hypertension had the highest population attributed risk (21.62%) followed by hypercholesterolemia (16.69%), diabetes (13.87%), low HDL-C (12.32%) and smoking (7.57%). As for all-cause mortality, having less than 6 years of education had the highest attributed risk (27.08%), followed by diabetes (24.37%), hypertension (17.13%), prevalent CVD (8.12%) and smoking (7.71).

Multivariate-adjusted hazard ratios and 95% confidence intervals (95% CIs) of potential cardiovascular risk factors as well as their PAFs for incident CVD and all-cause mortality, controlled for central adiposity and in the absence and presence of obesity mediators are shown in Tables A and B in S1 File, respectively. Results were generally as same as those observed in the presence of general adiposity; however, in contrast to general adiposity, abdominal obesity had a significant association with CVD, in the presence of mediators (1.17, 1.01–1.35).

The results of imputation analysis were similar to our main results (Tables C-J in S1 File).

## Discussion

Using data from a 10-year follow-up of an Iranian adult population, we estimated the association of potential cardiovascular risk factors as well as their PAFs for incident CVD and all-cause mortality rate. To the best of our knowledge, this is the first study conducted in the Middle East region, reporting the population attributable fraction of potential risk factors for incident CVD and all-cause mortality. Considering CVD event, in the absence of obesity mediators, general obesity, being overweight and high WC was associated with higher CVD incidence. Additionally, in the presence of mediators, hypercholesterolemia, low HDL-C, hypertension, diabetes, and smoking were attributed to higher CVD event rate. Central obesity, per se, in the presence of obesity mediators, had an independent positive association with CVD incidence. Considering all-cause mortality, although significant positive associations were observed for hypertension, diabetes, smoking and low education level; hypertriglyceridemia was inversely associated. Modifiable risk factors including hypertension, diabetes, smoking (for both CVD and mortality events), plus hypercholesteremia and low HDL-C (for CVD) and low education level (for mortality events) accounted for more than 70% of the PAF of both outcomes.

General and central obesity which are commonly assessed with BMI and WC respectively were associated with higher risk of incident CVD and all-cause mortality [32, 33]. In the current study, only in the absence of obesity mediators, being overweight and obesity showed significant risk for CVD events and whereas all together they showed a PAF of about 23%. However, in our data analysis, it has been indicated that the detrimental effect of general obesity on CVD event was mediated by hypertension, T2D and serum lipid profile. A pooled analysis of 97 prospective cohorts with 1.8 million participants, showed almost over 50% of the excess risk for CVD was mediated through three metabolic risk factors which can be explained as obesity mediators i.e. blood pressure categories, glucose intolerance status, abnormal lipid profile [24]. Furthermore, studies showed that obesity is associated with an increased incidence of CKD [25, 26] and CKD, per se, plays a role as an independent predictor of MI, stroke, and death [27, 28]. Hence, we added CKD as another obesity mediator.

In line with other studies [34–36], we showed that central adiposity was associated with higher risk of incident CVD. A finding which indicated that, in contrast to BMI, in the presence of obesity mediators, WC, per se, increased the risk of CVD incidence. In several large-scale studies, WC was found to be one of the most important predictors of CVD [37–39]. Cerhan.et al, also reported higher WC in both genders to be associated with higher mortality at all levels of BMI between 20–50 kg/m^2^ [34]; their finding supports the importance and major role of central obesity over general obesity in predicting incident CVD; which also was highlighted in other publications[40, 41]. On the other hand, for all-cause mortality, consistent with several studies, our results are supportive of the well-known “obesity paradox” [42–44]. We found that compared to normal BMI participants, overweight and obsess individuals had over 20% decreased risk for all-cause mortality. It has been shown that there is a U-shaped association between BMI and all-cause mortality with the concave regions sitting in the region of BMI 22–27 kg/m^2^[45]; this U-shaped relationship might be a result of the fact that BMI is composed of different components, i.e. fat mass, and fat-free mass, which have opposite effects on mortality, i.e. in contrast to fat mass, fat-free mass has an inverse association with mortality [46]. Furthermore, in a recent meta-analysis, overweight and moderately obese participants had lower mortality compared to normal BMI participants [33]. The authors concluded that use of predefined standard BMI groupings can facilitate between-study comparisons. Hence in the current study, we applied standard cutoffs for categorization of general adiposity. It should also be considered that over half of obese subjects in our population had grade 1 obesity (30–35 kg/m^2^) (Data not shown). Possible explanations include the earlier presentation of illness in overweight/obese population, greater likelihood of receiving optimal medical treatment, the cardio-protective metabolic effects of increased body fat, having better nutrition status and benefits of higher metabolic reserves [47]. It also should be considered in our study, following CVD, cancer and sepsis were the most common causes of death and weight loss is one of the most well-known poor prognostic factors in patients with both malignant and inflammatory diseases [48–50].

In line with previous studies, we observed both incident CVD and all-cause mortality to be increased among participants with diabetes [51–53]. Generally, diabetes is a prevalent metabolic disorder among Iranians [54] and is known as a coronary heart disease equivalent [55]. In the light of the high incidence and PAF of diabetes in this population [56], greater fraction of CVD and mortality event rates are expected to be attributable to diabetes in the future.

Similar to previous studies, we showed that hypertension is an independent, putative risk factor for incident CVD and all-cause mortality [57] and reduction in hypertension incidence remarkably reduces the risk of incident CVD and all-cause. In fact, hypertension is known as the most important modifiable risk factor for CVD and all-cause mortality worldwide [58, 59]. Therefore, more aggressive efforts may help decrease CVD risk and mortality among those already affected by inadequately treated or untreated hypertension.

More than 7% of incident CVD and of all-cause mortality were attributable to smoking in our study sample. Compared to current literature which reported smoking to be responsible for approximately 12% and 6% of male and female deaths respectively, worldwide [60, 61]. Recent studies also show that smoking in Iranian populations has an experiencing increasing trend [10, 62, 63]; this coupled with the fact that in Iran, unlike developed countries, smoking cessation counseling services are infrequently available or actively offered by health professionals.

It has been reported that even smoking a few cigarettes during childhood are associated with significantly higher odds of daily smoking in adolescence [64]. The prevalence of 15% was reported for current smoking in a study conducted among Iranian male high school adolescents [65]. However, unfortunately, we have no data in our study sample about the initiation trend of smoking and amount of tobacco use during childhood. Our findings showed that prioritization of smoking controlling programs at national levels to be effective strategies for the prevention of CVD and premature death.

Considering dyslipidemia, in the current study, hypercholesterolemia and low HDL-C, all together, account for about 30% of PAF of incident CVD. The significant roles of hypercholesterolemia and low HDL-C as well-established risk factors for incident CVD have been highlighted before[66]. Treatment of elevated cholesterol and mixed lipid disorders using statins may relieve some of the burden, as recently noted for patients with diabetes and stroke[66]. Despite the fact that the lipid-lowering drugs consumption in our population has increased remarkably (1.59 vs. 6% within a decade)[10], almost one-third of our population with dyslipidemia have not achieved the target levels of lipid measures may be due to poor compliance [10]. We also observed a favorable time trends in the population levels of lipid profile including HDL-C during 10 years follow-up [11].

One intriguing result reached through our study was that high serum TG was inversely associated with all-cause mortality among participants. Recently, we showed that 1 SD increment in log-transformed TGs was accompanied with 19% lower risk of non-CVD mortality [67]. A contention in contrast with previous studies, which highlighted the linear relation between hypertriglyceridemia and mortality [68, 69]; one explanation would be that low TG could be a marker of malnutrition and weight loss which, in turn, may increase mortality risk in the population [70]. In our study, after CVD, cancer was the most common cause of death and it has been shown that the low TG and weight loss are poor prognostic signs in cancerous patients and are associated with poor survival [48–50, 71]. Recently, we showed that hypertriglyceridemic waist circumference increased the age-adjusted risk of incident CVD, among both men and women [72]. Noticeably, in previous studies, we detected favorable time trends in the population levels of TG in the TLGS population (from 2.11 to 1.94 mmol/L in men and from 1.88 to 1.74 mmol/L in women) [11]; this significant decrease in TG levels could be the other plausible justification to this intriguing result.

Importantly, lower education level potentially increased the risk of all-cause mortality by about 60% and showed the highest PAF of 27% among other risk factors. A steady relationship documented between mortality and educational inequalities indicated that higher education status is associated with better adaptation to preventive lifestyles, lower prevalence of risk factors, early diagnosis and management of chronic disease risk factors, better quality treatment of acute diseases and lower risk of malnutrition and infection [73]. In line with our findings, Yusuf et al. found that nine easily measured and potentially modifiable risk factors such as education level account for an overwhelmingly large (over 90%) proportion of the risk of an initial acute myocardial infarction [74].

The strengths of our study include the prospective nature, the use of a large population-based-cohort of both sexes, accurate and valid data on risk factors at baseline and continuous surveillance of mortality, based on standard criteria. Our findings, however, need to be interpreted in the light of our study limitations. First, the population studied was of Persian ancestry, because of which, our results might not be directly extrapolated to other populations. Second, we only considered the status of risk factors at baseline and did not consider changes during the long-term follow-up. Third, we did not have enough statistical power to investigate the impact of risk factors for cause-specific mortality events. Fourth, we also used PAF as an epidemiologic tool to quantify the burden of CVD and mortality attributable to modifiable risk factors. This guides policymakers in prioritizing health strategies and interventions. In fact, PAF is a theoretical concept, and total elimination of certain risk factors by conducting intervention programs at the population level is generally almost impossible. In this regard, PAF is a better tool for prioritization than attributable risk. In most epidemiological studies, PAF measures only the excess fraction (the proportion of cases developing over some period of time among the exposed population, i.e. “excess” in comparison with the unexposed), rather than etiologic fraction (the proportion of cases in which the exposure has a causal role in disease occurrence) [75]. Last but not least, in a population-based observational cohort study of TLGS, the extent and importance of the risk factors are highlighted and the effect of treatment, interventions and the impact of different methods for accomplishing the changes shall be tested in clinical trial settings.

## Conclusion

Among modifiable risk factors, we found positive associations between diabetes, hypertension, current smoking and both outcomes; hypercholesterolemia, low HDL-C and CVD incidence; low education level and all-cause mortality events. These potentially modifiable risk factors which account for over 70% risk for both CVD and mortality events, indicating that modification of derived risk factors has the potential to prevent most CVD/all-cause mortality events among Tehranian adults.

## Supporting Information

S1 File Supporting Information file containing multiple supporting tables. Multivariate-adjusted hazard ratios and 95% confidence intervals (CIs) of potential cardiovascular risk factors as well as their PAFs for incident CVD and all-cause mortality, controlled for central adiposity and in the absence and present of obesity mediators (table A and B). Results from multivariate imputation by chained equation, using a regression including all of the variables in the main models (table C-J)
(PDF)

S2 File Data for mortality analysis (including individuals with CVD prevalence) (DTA)

S3 File Data for CVD analysis (DTA)
