# Supplementary material for: Risk Factors for Incidence of Cardiovascular Diseases and All-Cause Mortality in a Middle Eastern Population over a Decade Follow-up: Tehran Lipid and Glucose Study
Source: PLoS One. 2016 Dec 8;11(12):e0167623. doi: 10.1371/journal.pone.0167623 (PMC5145170; doi:10.1371/journal.pone.0167623)
Supplement: S1 File — Multivariate-adjusted hazard ratios and 95% confidence intervals (CIs) of potential cardiovascular risk factors as well as their PAFs for incident CVD and all-cause mortality, controlled for central adiposity and in the absence and present of obesity mediators (table A and B). Results from multivariate imputation by chained equation, using a regression including all of the variables in the main models (table C-J) (PDF) [file pone.0167623.s001.pdf]

Table A. Multivariate-adjusted hazard ratios and 95% confidence intervals (CIs) of potential cardiovascular risk factors for incident CVD and all-cause mortality, without obesity mediators controlling for central adiposity status, Tehran lipid and Glucose study (1999-2005)

	CVD event						All-cause mortality					
	HR	95% confidence interval	p-value	Prevalence *	95% confidence interval	PAF**	HR	95% confidence interval	p-value	Prevalence *	95% confidence interval	PAF**
Central obesity	1.49	1.30-1.71	<0.0001	0.52	0.48-0.55	17.10	1.09	0.92-1.29	0.292			
Current Smoker	1.51	1.25-1.81	<0.0001	0.20	0.17-0.23	6.75	1.67	1.33-2.11	<0.0001	0.18	0.14-0.21	7.22
Education												
>12 years	1						1			0.05	0.02-0.06	
6-12 years	1.22	0.92-1.62	0.161			-	1.50	0.94-2.38	0.087	0.22	0.19-0.260	7.33
<6 years	1.14	0.85-1.51	0.366			-	1.65	1.04-2.60	0.030	0.73	0.69-0.77	28.75
Family history of CVD	1.58	1.34-1.88	<0.0001	0.21	0.17-0.23	7.70	1.32	1.05-1.64	0.014	0.18	0.14-0.21	4.36
Male gender (female as a reference)	1.65	1.41-1.93	<0.0001			-	1.42	1.17-1.71	<0.0001			-
Prevalent CVD						-	1.97	1.59-2.42	<0.0001	0.21	0.17-0.23	9.70

*Prevalence represents the prevalence of the exposure among individuals with outcome of interest
**The population attributed fractions were calculated using the following formula: Prevalence*((Hazard ratio-1)/Hazard ratio)
HR, hazard ratio; HDL-C, high density lipoprotein cholesterol; CKD, chronic kidney disease;
The definition for categorical variables is shown in table 1.

Table B: Multivariate-adjusted hazard ratios and 95% confidence intervals (CIs) of potential cardiovascular risk factors for incident CVD and all-cause mortality, with obesity mediators (diabetes, hypertension, lipid profile and CKD), controlling for central adiposity status, Tehran lipid and Glucose study (1999-2005)

	CVD event							All-cause mortality						
	N(event)	HR	95% confidence interval	p-value	Prevalence *	95% confidence interval	PAF**	N(event)	HR	95% confidence interval	p-value	Prevalence *	95% confidence interval	PAF**
Central obesity	432	1.17	1.01-1.35	0.032	0.52	0.48-0.55	14.52		0.95	0.80-1.14	0.633			-
High TG	540	1.01	0.86-1.19	0.879				295	0.80	0.66-0.97	0.027	0.53	0.49-0.57	-13.25
High TC	346	1.58	1.36-1.84	<0.0001	0.45	0.51-0.58	16.51	191	1.03	0.85-1.25	0.726			
Low HDL-C	584	1.20	1.02-1.41	0.023	0.71	0.67-0.73	11.83	336	0.94	0.78-1.12	0.500			
Blood pressure status														
Normal	174	1			0.21	0.18-0.23		105	1			0.20	0.15-0.22	
prehypertension	241	1.12	0.98-1.37	0.260	0.29	0.26-0.32		129	0.86	0.66-1.13	0.297	0.23	0.19-0.26	
Hypertension	412	1.75	1.43-2.14	<0.0001	0.49	0.46-0.53	21.00	317	1.36	1.06-1.74	0.014	0.57	0.53-0.61	15.08
Blood sugar status														
Normal	353	1			0.43	0.39-0.46		200	1			0.36	0.32-0.40	
prediabetes	223	1.10	0.92-1.31	0.264	0.27	0.23-0.29		130	1.10	0.88-1.39	0.368	0.23	0.20-0.27	
Diabetes	251	1.83	1.53-2.18	<0.0001	0.30	0.27-0.33	13.60	221	2.48	2.02-3.05	<0.0001	0.40	0.36-0.44	23.87
CKD	276	1.02	0.87-1.19	0.835			-	233	1.00	0.83-1.21	0.981			
Current Smoker	168	1.61	1.33-1.93	<0.0001	0.20	0.17-0.23	7.57	100	1.81	1.42-2.28	<0.0001	0.18	0.14-0.21	8.05
Education														
>12 years	59	1						21	1			0.05	0.02-0.06	
6-12 years	269	1.19	0.89-1.58	0.225			-	124	1.48	0.93-2.36	0.094	0.22	0.19-0.260	7.13
<6 years	499	1.11	0.84-1.48	0.464			-	406	1.59	1.01-2.51	0.045	0.73	0.69-0.77	27.08
Family history of CVD	171	1.52	1.28-1.81	<0.0001	0.21	0.17-0.23	7.18	99	1.22	0.98-1.53	0.07	0.18	0.14-0.21	3.24
Male gender(female as a reference)	490	2.02	1.71-2.38	<0.0001			-	340	1.49	1.22-1.83	<0.0001			-
Prevalent CVD	-						-	472	1.62	1.31-2.01	<0.0001	0.21	0.17-0.23	8.04

*Prevalence represents the prevalence of the exposure among individuals with outcome of interest
**The population attributed fractions were calculated using the following formula: Prevalence*((Hazard ratio -1)/Hazard ratio)
HR, hazard ratio; HDL-C, high density lipoprotein cholesterol; CKD, chronic kidney disease;
The definition for categorical variables is shown in table 1.

Table C. Multivariate-adjusted hazard ratios and 95% confidence intervals (CIs) of potential cardiovascular risk factors for incident CVD, without obesity mediators controlling for general adiposity status using single imputation method, Tehran lipid and Glucose study (1999-2012)

	event	HR	HR (95%CI)	p-value	Prevalence*	Prevalence (95%CI)	PAF (%)**
BMI							
Normal	222	1			0.25	0.22 - 0.28	
Overweight	414	1.4	1.18 - 1.65	<0.0001	0.46	0.43 - 0.5	13.14
Obesity	257	1.55	1.28 - 1.87	<0.0001	0.29	0.26 - 0.32	10.29
Current Smoker	189	1.53	1.29 - 1.82	<0.0001	0.21	0.18 - 0.24	7.27
Education							
>12 years	66	1					
6-12 years	291	1.21	0.93 - 1.59	0.158			
<6 years	536	1.17	0.89 - 1.53	0.266			
Family history of CVD	182	1.57	1.33 - 1.85	<0.0001	0.2	0.18 - 0.23	7.26
Male gender (female as a reference)	537	1.78	1.53 - 2.07	<0.0001			

*Prevalence represents the prevalence of the exposure among individuals with outcome of interest

**The population attributed fractions were calculated using the following formula: Prevalence*((Hazard ratio-1)/Hazard ratio)

HR, hazard ratio; HDL-C, high density lipoprotein cholesterol; CKD, chronic kidney disease;

The definition for categorical variables is shown in table 1.

Table D. Multivariate-adjusted hazard ratios and 95% confidence intervals (CIs) of potential cardiovascular risk factors for incident CVD, without obesity mediators controlling for central adiposity status using single imputation method, Tehran lipid and Glucose study (1999-2012)

	event	HR	HR (95%CI)	p-value	Prevalence*	Prevalence (95%CI)	PAF (%)**
Central obesity	462	1.48	1.29 - 1.69	<0.0001	0.52	0.48 - 0.55	16.86
Current Smoker	189	1.5	1.26 - 1.78	<0.0001	0.21	0.18 - 0.24	7
Education	66	1					
>12 years	291	1.21	0.92 - 1.58	0.165			
6-12 years	536	1.15	0.87 - 1.5	0.324			
<6 years	182	1.57	1.33 - 1.85	<0.0001			
Family history of CVD	537	1.69	1.45 - 1.96	<0.0001	0.2	0.18 - 0.23	7.26
Male gender (female as a reference)	462	1.48	1.29 - 1.69	<0.0001			

*Prevalence represents the prevalence of the exposure among individuals with outcome of interest

**The population attributed fractions were calculated using the following formula: Prevalence*((Hazard ratio-1)/Hazard ratio)

HR, hazard ratio; HDL-C, high density lipoprotein cholesterol; CKD, chronic kidney disease;

The definition for categorical variables is shown in table 1.

Table E. Multivariate-adjusted hazard ratios and 95% confidence intervals (CIs) of potential cardiovascular risk factors for incident CVD, with obesity mediators (diabetes, hypertension, lipid profile and CKD), controlling for general adiposity status, using single imputation method, Tehran lipid and Glucose study (1999-2005)

	event	HR	HR (95%CI)	p-value	Prevalence*	Prevalence (95%CI)	PAF (%)**
BMI							
Normal	222	1					
Overweight	414	1.15	0.97 - 1.36	0.109			
Obesity	257	1.09	0.9 - 1.33	0.38			
High TG	578	1.01	0.86 - 1.18	0.936		0.62 - 0.68	
High TC	397	1.59	1.37 - 1.83	<0.0001	0.44	0.41 - 0.48	16.33
low HDL-C	633	1.22	1.05 - 1.42	0.011	0.71	0.68 - 0.74	12.8
Blood pressure status							
Normal	190	1			0.21	0.19 - 0.24	
prehypertension	259	1.12	0.92 - 1.36	0.259	0.29	0.26 - 0.32	
Hypertension	444	1.81	1.5 - 2.2	<0.0001	0.5	0.46 - 0.53	22.38
Blood sugar status							
Normal	391	1			0.44	0.41 - 0.47	
Pre diabetes	233	1.11	0.94 - 1.31	0.218	0.26	0.23 - 0.29	
Diabetes	269	1.88	1.59 - 2.22	<0.0001	0.3	0.27 - 0.33	14.04
CKD	295	1.03	0.88 - 1.2	0.698			
Current Smoker	189	1.63	1.37 - 1.95	<0.0001	0.21	0.18 - 0.24	8.12
Education							
>12 years	66	1					
6-12 years	291	1.17	0.9 - 1.53	0.248			
<6 years	536	1.13	0.86 - 1.48	0.395			
Family history of CVD	182	1.51	1.28 - 1.78	<0.0001	0.2	0.18 - 0.23	6.75
Male gender (female as a reference)	537	2.09	1.78 - 2.45	<0.0001			

*Prevalence represents the prevalence of the exposure among individuals with outcome of interest

**The population attributed fractions were calculated using the following formula: Prevalence*((Hazard ratio-1)/Hazard ratio)

HR, hazard ratio; HDL-C, high density lipoprotein cholesterol; CKD, chronic kidney disease;

The definition for categorical variables is shown in table 1.

Table F. Multivariate-adjusted hazard ratios and 95% confidence intervals (CIs) of potential cardiovascular risk factors for incident CVD, with obesity mediators (diabetes, hypertension, lipid profile and CKD), controlling for general adiposity status, using single imputation method, Tehran lipid and Glucose study (1999-2005)

	event	HR	HR (95%CI)	p-value	Prevalence*	Prevalence (95%CI)	PAF (%)**
Central obesity	462	1.16	1.01 - 1.33	0.041	0.52	0.48 - 0.55	7.17
High TG	578	1	0.86 - 1.17	0.983			
High TC	397	1.58	1.37 - 1.83	<0.0001	0.44	0.41 - 0.48	16.15
low HDL-C	633	1.21	1.04 - 1.42	0.013	0.71	0.68 - 0.74	12.32
Blood pressure status							
Normal	190	1			0.21	0.19 - 0.24	
prehypertension	259	1.12	0.92 - 1.35	0.258	0.29	0.26 - 0.32	
Hypertension	444	1.79	1.48 - 2.17	<0.0001	0.5	0.46 - 0.53	22.07
Blood sugar status							
Normal	391	1			0.44	0.41 - 0.47	
Pre diabetes	233	1.1	0.93 - 1.3	0.261	0.26	0.23 - 0.29	
Diabetes	269	1.85	1.57 - 2.19	<0.0001	0.3	0.27 - 0.33	13.78
CKD	295	1.03	0.88 - 1.2	0.689			
Current Smoker	189	1.62	1.36 - 1.93	<0.0001	0.21	0.18 - 0.24	8.04
Education							
>12 years	66	1					
6-12 years	291	1.17	0.89 - 1.53	0.258			
<6 years	536	1.11	0.85 - 1.46	0.448			
Family history of CVD	182	1.51	1.28 - 1.78	<0.0001	0.2	0.18 - 0.23	6.75
Male gender (female as a reference)	537	2.07	1.77 - 2.43	<0.0001			

*Prevalence represents the prevalence of the exposure among individuals with outcome of interest

**The population attributed fractions were calculated using the following formula: Prevalence*((Hazard ratio-1)/Hazard ratio)

HR, hazard ratio; HDL-C, high density lipoprotein cholesterol; CKD, chronic kidney disease;

The definition for categorical variables is shown in table 1.

Table G. Multivariate-adjusted hazard ratios and 95% confidence intervals (CIs) of potential cardiovascular risk factors for all-cause mortality, without obesity mediators controlling for general adiposity status using single imputation method, Tehran lipid and Glucose study (1999-2012)

	event	HR	HR (95%CI)	p-value	Prevalence*	Prevalence (95%CI)	PAF (%)**
BMI							
Normal	220	1			0.36	0.32 - 0.4	
Overweight	241	0.83	0.69 - 1	0.05	0.4	0.36 - 0.44	
Obesity	145	1.02	0.81 - 1.28	0.866	0.24	0.21 - 0.27	
Current Smoker	114	1.64	1.32 - 2.04	<0.0001	0.19	0.16 - 0.22	7.41
Education							
>12 years	23	1			0.04	0.02 - 0.05	
6-12 years	135	1.59	1.02 - 2.48	0.04	0.22	0.19 - 0.26	8.16
<6 years	448	1.8	1.17 - 2.77	0.008	0.74	0.7 - 0.77	32.89
Family history of CVD	105	1.33	1.08 - 1.65	0.009	0.17	0.14 - 0.2	4.22
Male gender (female as a reference)	377	1.48	1.23 - 1.78	<0.0001			
Prevalence CVD	130	2.03	1.67 - 2.48	<0.0001	0.21	0.18 - 0.25	10.66

*Prevalence represents the prevalence of the exposure among individuals with outcome of interest

**The population attributed fractions were calculated using the following formula: Prevalence*((Hazard ratio-1)/Hazard ratio)

HR, hazard ratio; HDL-C, high density lipoprotein cholesterol; CKD, chronic kidney disease;

The definition for categorical variables is shown in table 1.

Table H. Multivariate-adjusted hazard ratios and 95% confidence intervals (CIs) of potential cardiovascular risk factors for all-cause mortality, without obesity mediators controlling for central adiposity status using single imputation method, Tehran lipid and Glucose study (1999-2012)

	event	HR	HR (95%CI)	p-value	Prevalence*	Prevalence (95%CI)	PAF (%)**
Central obesity	279	1.1	0.94 - 1.3	0.242			
Current Smoker	114	1.67	1.34 - 2.07	<0.0001	0.19	0.16 - 0.22	7.62
Education							
>12 years	23	1			0.04	0.02 - 0.05	
6-12 years	135	1.59	1.02 - 2.47	0.041	0.22	0.19 - 0.26	8.16
<6 years	448	1.77	1.15 - 2.73	0.009	0.74	0.7 - 0.77	32.19
Family history of CVD	105	1.33	1.07 - 1.64	0.01	0.17	0.14 - 0.2	4.22
Male gender (female as a reference)	377	1.47	1.23 - 1.77	<0.0001			
Prevalence CVD	130	2	1.64 - 2.44	<0.0001	0.21	0.18 - 0.25	10.5

*Prevalence represents the prevalence of the exposure among individuals with outcome of interest

**The population attributed fractions were calculated using the following formula: Prevalence*((Hazard ratio-1)/Hazard ratio)

HR, hazard ratio; HDL-C, high density lipoprotein cholesterol; CKD, chronic kidney disease;

The definition for categorical variables is shown in table 1.

Table I. Multivariate-adjusted hazard ratios and 95% confidence intervals (CIs) of potential cardiovascular risk factors for all-cause mortality, with obesity mediators (diabetes, hypertension, lipid profile and CKD), controlling for general adiposity status, using single imputation method, Tehran lipid and Glucose study (1999-2005)

	event	HR	HR (95%CI)	p-value	Prevalence *	Prevalence (95%CI)	PAF (%)**
BMI							
Normal	220	1			0.36	0.32 - 0.4	
Overweight	241	0.73	0.6 - 0.89	0.002	0.4	0.36 - 0.44	-14.79
Obesity	145	0.81	0.64 - 1.02	0.07	0.24	0.21 - 0.27	-5.62
High TG	321	0.83	0.69 - 0.99	0.041	0.53	0.49 - 0.57	-10.86
High TC	230	1.05	0.87 - 1.25	0.634	0.38	0.34 - 0.42	
low HDL-C	377	0.99	0.83 - 1.18	0.9	0.62	0.58 - 0.66	
Blood pressure status							
Normal	113	1			0.19	0.16 - 0.22	
prehypertension	147	0.98	0.76 - 1.27	0.896	0.24	0.21 - 0.28	
Hypertension	346	1.49	1.18 - 1.9	0.001	0.57	0.53 - 0.61	18.74
Blood sugar status							
Normal	229	1			0.38	0.34 - 0.42	
Pre diabetes	141	1.14	0.92 - 1.42	0.233	0.23	0.2 - 0.27	
Diabetes	236	2.53	2.07 - 3.08	<0.0001	0.39	0.35 - 0.43	23.58
CKD	256	1.03	0.86 - 1.23	0.749	0.42	0.38 - 0.46	
Current Smoker	114	1.8	1.44 - 2.25	<0.0001	0.19	0.16 - 0.22	8.44
Education							
>12 years	23	1			0.04	0.02 - 0.05	
6-12 years	135	1.54	0.99 - 2.4	0.056	0.22	0.19 - 0.26	
<6 years	448	1.69	1.1 - 2.61	0.017	0.74	0.7 - 0.77	30.21
Family history of CVD	105	1.23	0.99 - 1.53	0.057	0.17	0.14 - 0.2	
Male gender (female as a reference)	377	1.53	1.26 - 1.86	<0.0001			
Prevalent CVD	130	1.67	1.36 - 2.05	<0.0001	0.21	0.18 - 0.25	8.43

*Prevalence represents the prevalence of the exposure among individuals with outcome of interest

**The population attributed fractions were calculated using the following formula: Prevalence*((Hazard ratio-1)/Hazard ratio)

HR, hazard ratio; HDL-C, high density lipoprotein cholesterol; CKD, chronic kidney disease;

The definition for categorical variables is shown in table 1.

Table J. Multivariate-adjusted hazard ratios and 95% confidence intervals (CIs) of potential cardiovascular risk factors for all-cause, with obesity mediators (diabetes, hypertension, lipid profile and CKD), controlling for general adiposity status, using single imputation method, Tehran lipid and Glucose study (1999-2005)

	event	HR	HR (95%CI)	p-value	Prevalence*	Prevalence (95%CI)	PAF (%)**
Central obesity	279	0.95	0.81 - 1.13	0.588			
High TG	321	0.8	0.67 - 0.96	0.017	0.53	0.49 - 0.57	-13.25
High TC	230	1.04	0.87 - 1.25	0.667			
low HDL-C	377	0.98	0.82 - 1.17	0.844			
Blood pressure status							
Normal	113	1			0.19	0.16 - 0.22	
prehypertension	147	0.93	0.73 - 1.2	0.601	0.24	0.21 - 0.28	
Hypertension	346	1.43	1.13 - 1.81	0.003	0.57	0.53 - 0.61	17.14
Blood sugar status							
Normal	229	1			0.38	0.34 - 0.42	
Pre diabetes	141	1.11	0.9 - 1.38	0.335	0.23	0.2 - 0.27	
Diabetes	236	2.45	2.01 - 2.99	<0.0001	0.39	0.35 - 0.43	23.08
CKD	256	1.01	0.85 - 1.21	0.895			
Current Smoker	114	1.84	1.48 - 2.3	<0.0001	0.19	0.16 - 0.22	8.67
Education							
>12 years	23	1			0.04	0.02 - 0.05	
6-12 years	135	1.54	0.99 - 2.41	0.055	0.22	0.19 - 0.26	7.71
<6 years	448	1.68	1.09 - 2.59	0.019	0.74	0.7 - 0.77	29.95
Family history of CVD	105	1.22	0.99 - 1.52	0.067			
Male gender (female as a reference)	377	1.54	1.27 - 1.87	<0.0001			
Prevalent CVD	130	1.66	1.36 - 2.04	<0.0001	0.21	0.18 - 0.25	8.35

*Prevalence represents the prevalence of the exposure among individuals with outcome of interest

**The population attributed fractions were calculated using the following formula: Prevalence*((Hazard ratio-1)/Hazard ratio)

HR, hazard ratio; HDL-C, high density lipoprotein cholesterol; CKD, chronic kidney disease;

The definition for categorical variables is shown in table 1
